# Laparoscopic segmental colectomy with extensive D3 lymph node dissection: a good choice for right transverse colon cancer

**DOI:** 10.1186/s12957-022-02530-4

**Published:** 2022-03-15

**Authors:** Xing Huang

**Affiliations:** The First Department of General Surgery (Department of Colorectal and Anal Surgery), Hunan Provincial People’s Hospital (The First Affiliated Hospital of Hunan Normal University), No. 61 Jiefang West Road, Changsha, Hunan China

**Keywords:** Transverse colon cancer (TCC), Segmental colectomy, Right hemicolectomy, D3 lymph node dissection

## Abstract

**Background:**

Previous research was yet to establish a definite operation for transverse colon cancer (TCC); surgical procedure was often dictated by the surgeon’s preference in clinical practice. The main surgical methods could be summarized in two main points: segmental colectomy (transverse colectomy) and right hemicolectomy.

**Method:**

The first patient was a 78-year-old woman, who was diagnosed with right TCC. Computed tomography revealed a right TCC and a very long transverse colon; laparoscopic exploration revealed an enlarged apical lymph node surrounding the ileocolic vessels. We performed a segmental colectomy with extensive apical lymph node dissection along the superior mesenteric vessels and its main branches for her. To distinguish it from the previous radical operations for TCC, we called this operation a segmental colectomy with extensive D3 lymph node dissection. Then, this surgical intervention was performed on 8 other TCC patients.

**Results:**

The total operating time was 158 min. Pathological examination confirmed 2 apical lymph node metastases; among them, one apical lymph node metastasis was in group No.203. For all 9 patients, the median operative time was 160 min (range, 140–185 min), the average number of lymph node retrieval was 30 (range, 25–39), and the average number of apical lymph node (No.203, No.213, and No.223) retrieval was 5.9 (range, 0–11). Because of the preservation of the ileocecal junction and part of the ascending colon, all patients recovered uneventfully after surgery, and long-term diarrhea, water-electrolyte imbalance, and other Clavien–Dindo grade III or greater postoperative complications did not occur.

**Conclusions:**

Our procedure combined the advantages of segmental colectomy and right hemicolectomy and gave consideration to oncological and functional outcomes. It may be an optimal choice for TCC patients with a very long transverse colon and preoperative diagnosis of lymph node metastasis.

**Supplementary Information:**

The online version contains supplementary material available at 10.1186/s12957-022-02530-4.

## Introduction

Transverse colon cancer (TCC) refers to a tumor located between the hepatic and splenic flexures of the colon. Due to the anatomical complexity and lack of large-scale randomized controlled trials, it is a challenge to standardize TCC operation. The transverse colon is in close proximity to many upper abdominal vital structures, so it is also a challenge to perform a radical operation for TCC. Previous research is yet to establish a definite operation for TCC; surgical procedure is often dictated by the surgeon’s preference in clinical practice. The main surgical methods can be summarized in two main points: segmental colectomy and right hemicolectomy. The former leads to a shorter specimen length and fewer lymph nodes harvested. Although the latter leads to a longer specimen length and more lymph nodes harvested, it has a greater trauma and causes some functional damage [1].

## Method

**Fig. 1:**
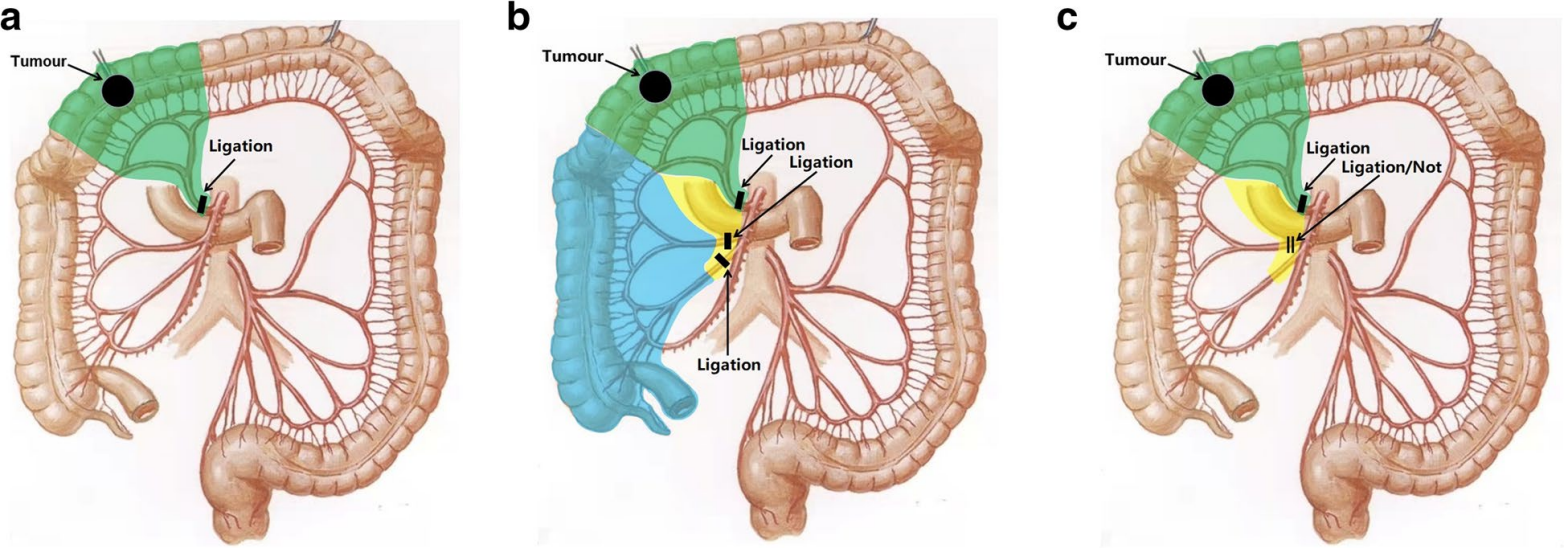
**A** Segmental colectomy with D3 lymph node dissection. **B** Right hemicolectomy with D3 lymph node dissection. **C** Segmental colectomy with extensive D3 lymph node dissection. Compared with operation A, an extensive apical lymph node dissection along the superior mesenteric vessels and its main branches is performed in operation C, while compared with operation B, the main vessels of the right-sided colon, ileocecal junction, and part of the ascending colon are preserved in operation C (if a RCA is too small, ligation is recommended). Anatomical pictures are from the *Atlas of Human Anatomy* [2]

**Fig. 2:**
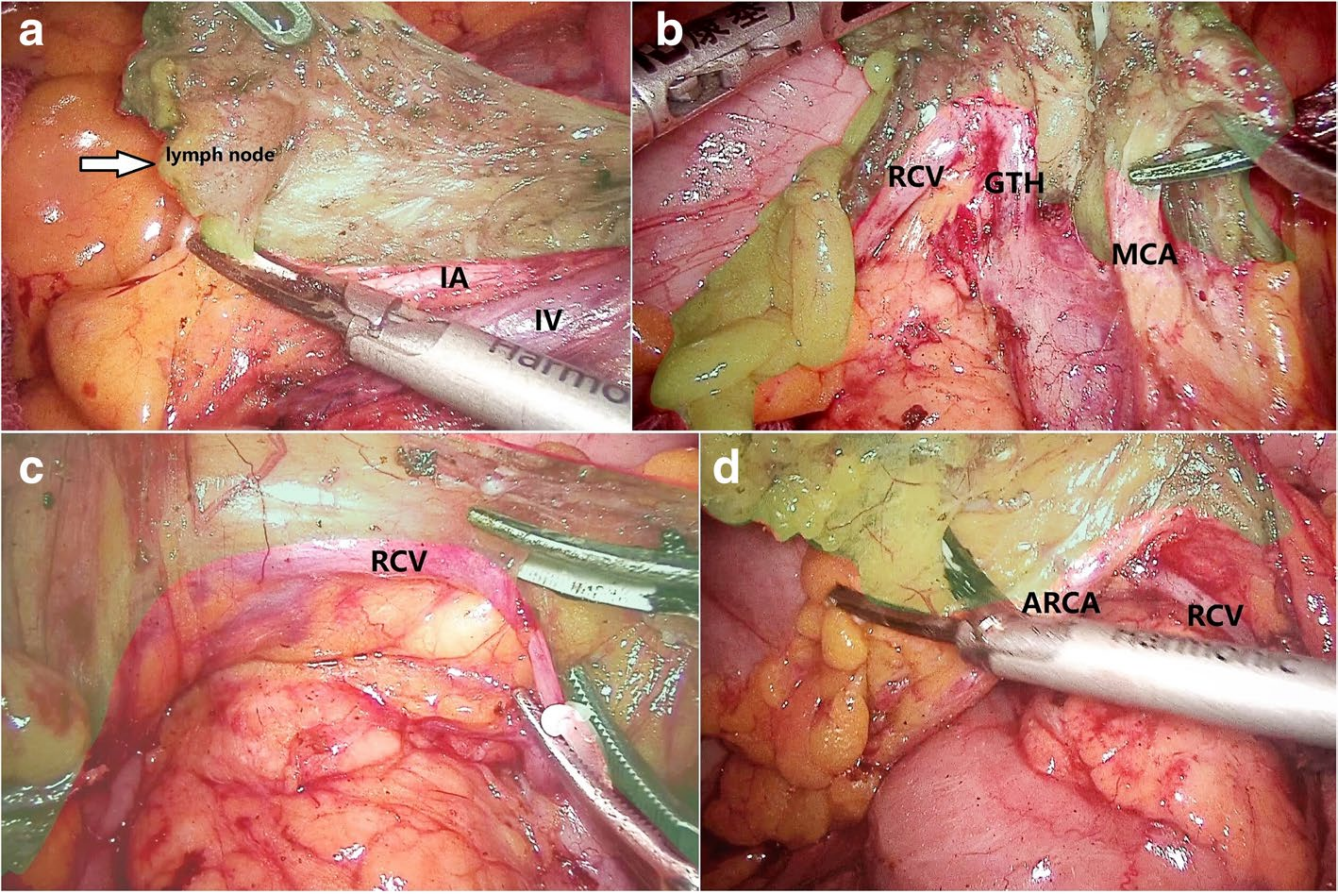
Compared with right hemicolectomy with D3 lymph node dissection, the main vessels of the right-sided colon should be preserved in our operation. **A** An enlarged apical lymph node surrounds the ileocolic vessels (arrow). Apical lymph node (no. 203) dissection is performed along the ileocolic vessels. **B** Apical lymph node (no. 223) dissection is performed along the middle colic artery (MCA). **C** The right colonic vein (RCV) is preserved, and a careful dissection along it is performed. **D** The accessory right colic artery (ARCA) is preserved, and a careful dissection along it is performed

**Fig. 3:**
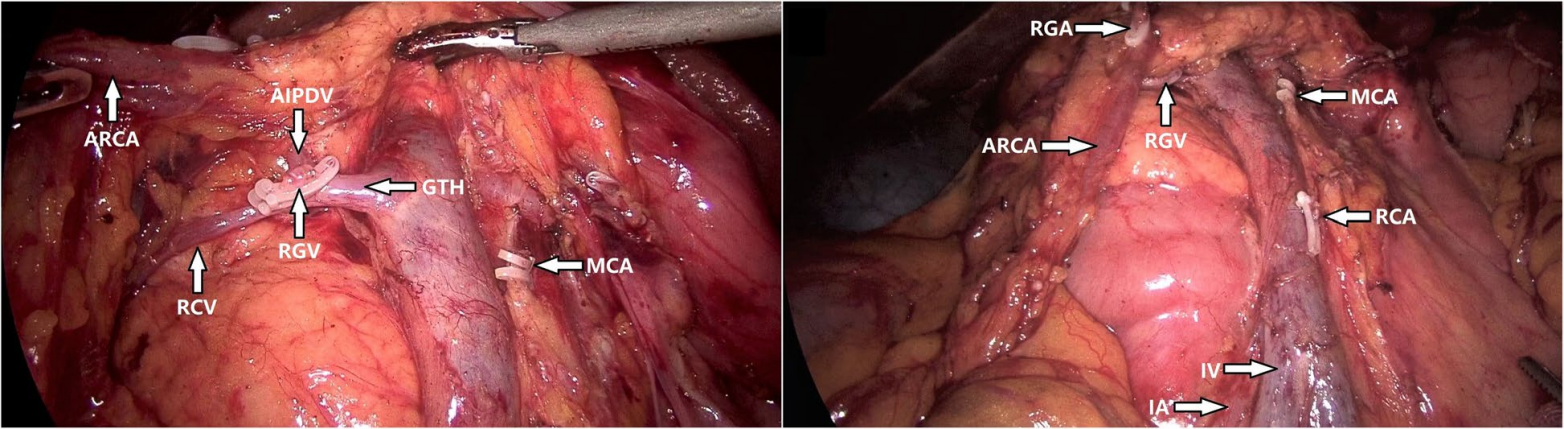
This is a surgical field after apical lymph node dissection. Compared with segmental colectomy with D3 lymph node dissection, an extensive apical lymph node dissection along the superior mesenteric vessels is performed; compared with right hemicolectomy with D3 lymph node dissection, IA, IV, ARCA, and RCV are preserved in our operation

**Fig. 4:**
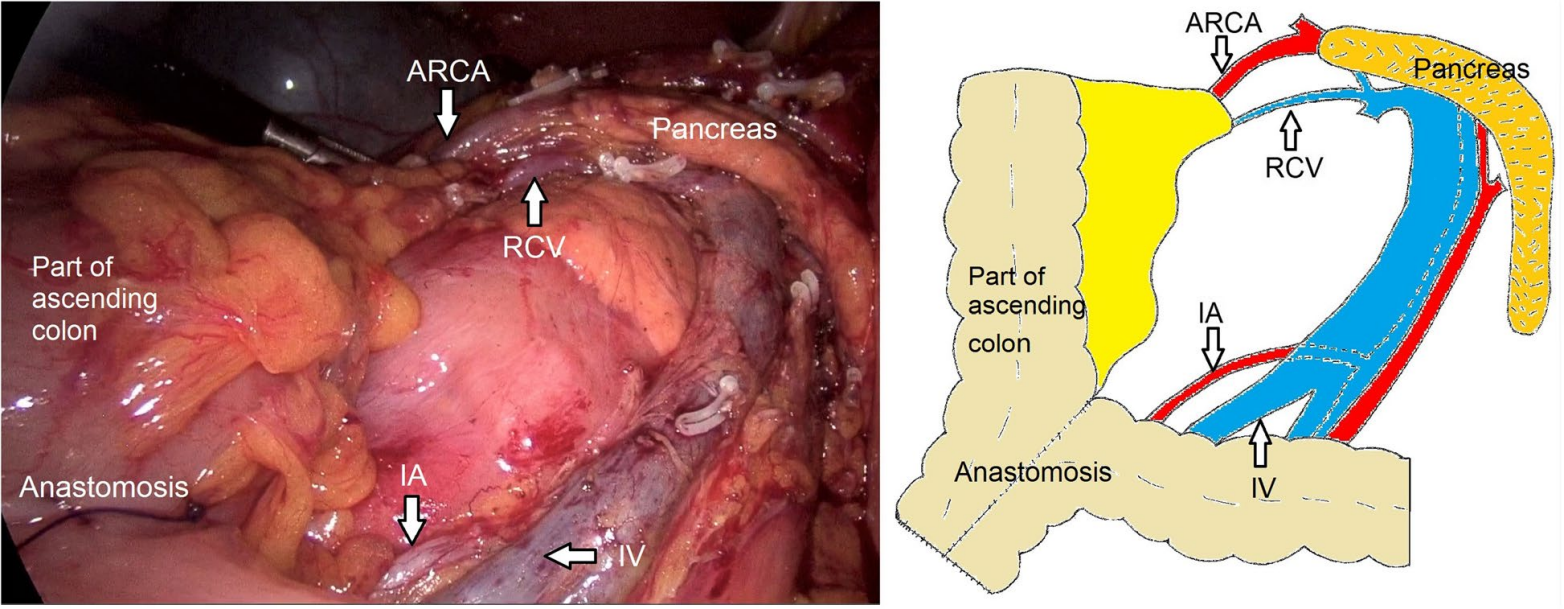
This is a surgical field after the operation. In this case, IA, IV, ARCA, RCV, ileocecal junction, and part of the ascending colon are preserved; at the same time, the apical lymph node (no. 203, No.223) dissection is performed

**Fig. 5:**
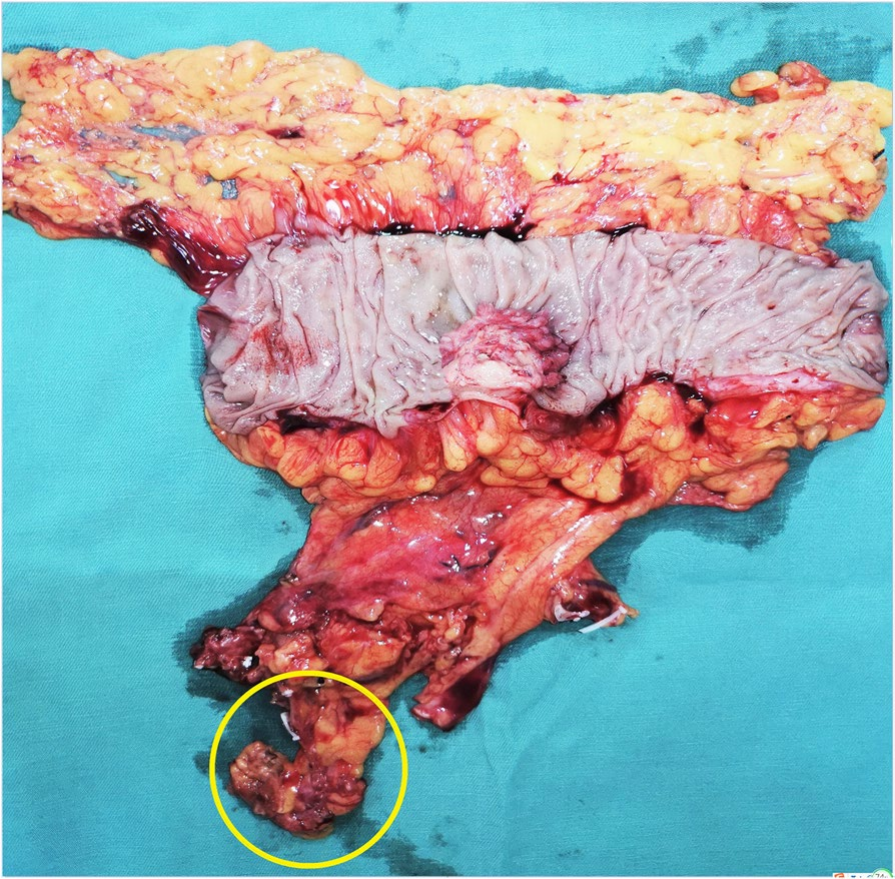
A specimen shows a complete mesorectal excision with extensive D3 lymph node dissection (yellow circle)

A 78-year-old woman presented with a 1-month history of blood-stained stool. Her medical history was notable for type 2 diabetes mellitus for 5 years, using insulin to control blood glucose. Computed tomography of the abdomen revealed wall thickening of the right transverse colon and a very long transverse colon but did not reveal obvious mesenteric lymphadenopathy, other organ involvement, and intestinal obstruction. Subsequently, a colonoscopy revealed a tumor in the right transverse colon, the distance from its distal margin to the anal verge was about 75 cm, and the pathological test indicated a moderately differentiated adenocarcinoma. Preoperative clinical staging was cT3N0M0. First, laparoscopic exploration revealed an enlarged apical lymph node surrounding the ileocolic vessels. Obviously, colectomy with D3 lymph node dissection was not enough for this situation. To avoid removing a long section of the colon in a right hemicolectomy, we planned to perform a segmental colectomy with extensive apical lymph node dissection along the superior mesenteric vessels and its main branches for her. To distinguish it from the previous radical operations for TCC, we called this operation a segmental colectomy with extensive D3 lymph node dissection. Compared with segmental colectomy with D3 lymph node dissection, a more extensive apical lymph node dissection was performed, and compared with right hemicolectomy with D3 lymph node dissection, the main vessels of the right-sided colon, ileocecal junction, and part of the ascending colon were preserved in our operation (Fig. 1). Key points of this operation included dissection of apical lymph nodes (No.203, No.213, No.223) and preservation of the main vessels of the right-sided colon. In this case, ileocolic artery (IA), ileocolic vein (IV), accessory right colic artery (ARCA), right colonic vein (RCV), ileocecal junction, and part of the ascending colon were preserved; at the same time, the apical lymph node (No.203, No.213, No.223) dissection was well performed (Figs. 2 and 3). Lastly, after a segment of the transverse colon with the tumor was removed, we performed a side-to-side intestinal anastomosis for her (Fig. 4). Her specimen showed a complete mesorectal excision with extensive D3 lymph node dissection (Fig. 5). A video demonstrating the laparoscopic segmental colectomy with extensive D3 lymph node dissection in right TCC was presented (Additional file 1: Supplementary video 1).

## Results

**Fig. 6:**
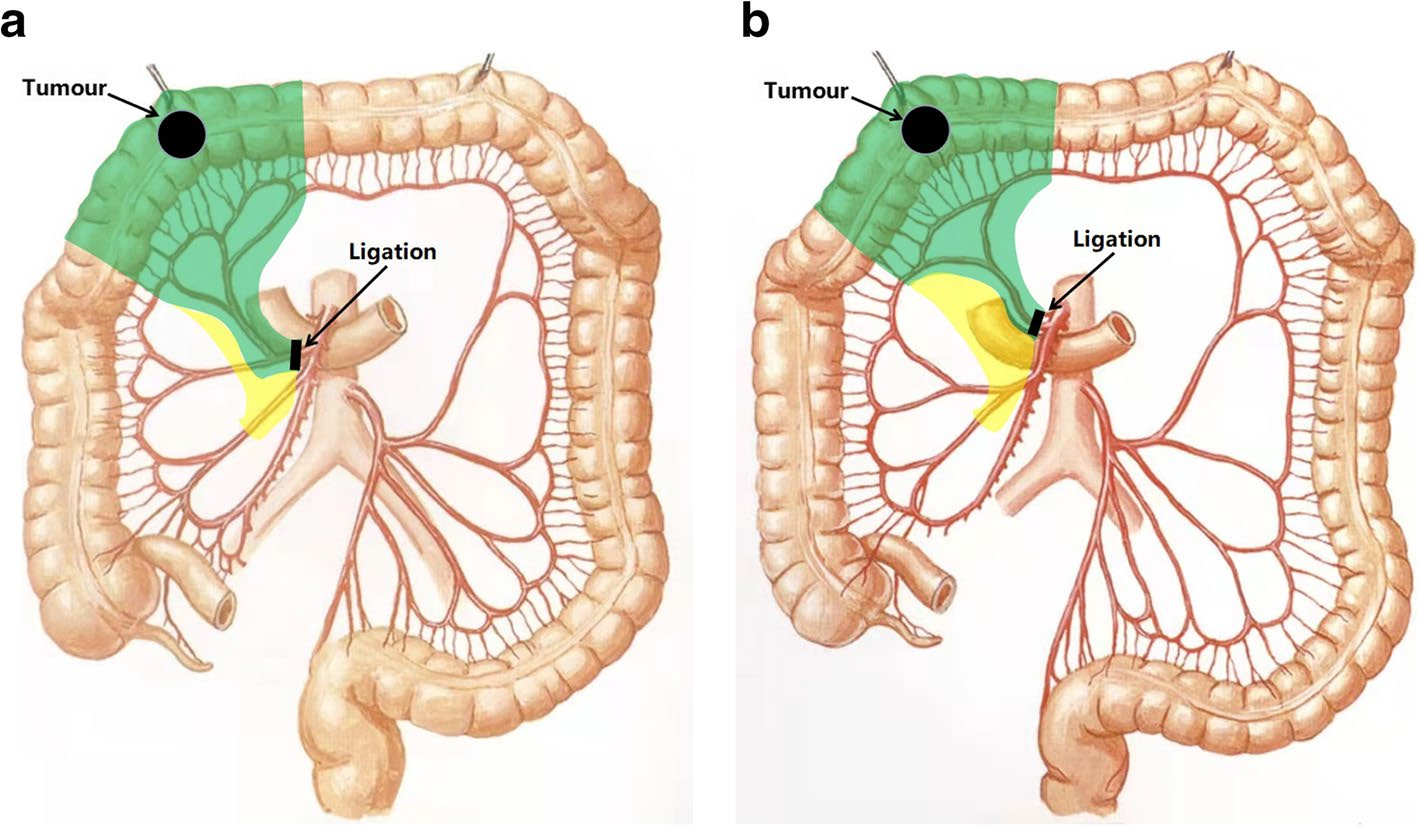
Vascular ligation locations and surgical resection ranges of our procedure for the two common patterns of common trunks. **A** Common trunks of RCA and MCA. **B** Common trunks of RCA and IA. Anatomical pictures are from the *Atlas of Human Anatomy* [2]

**Fig. 7:**
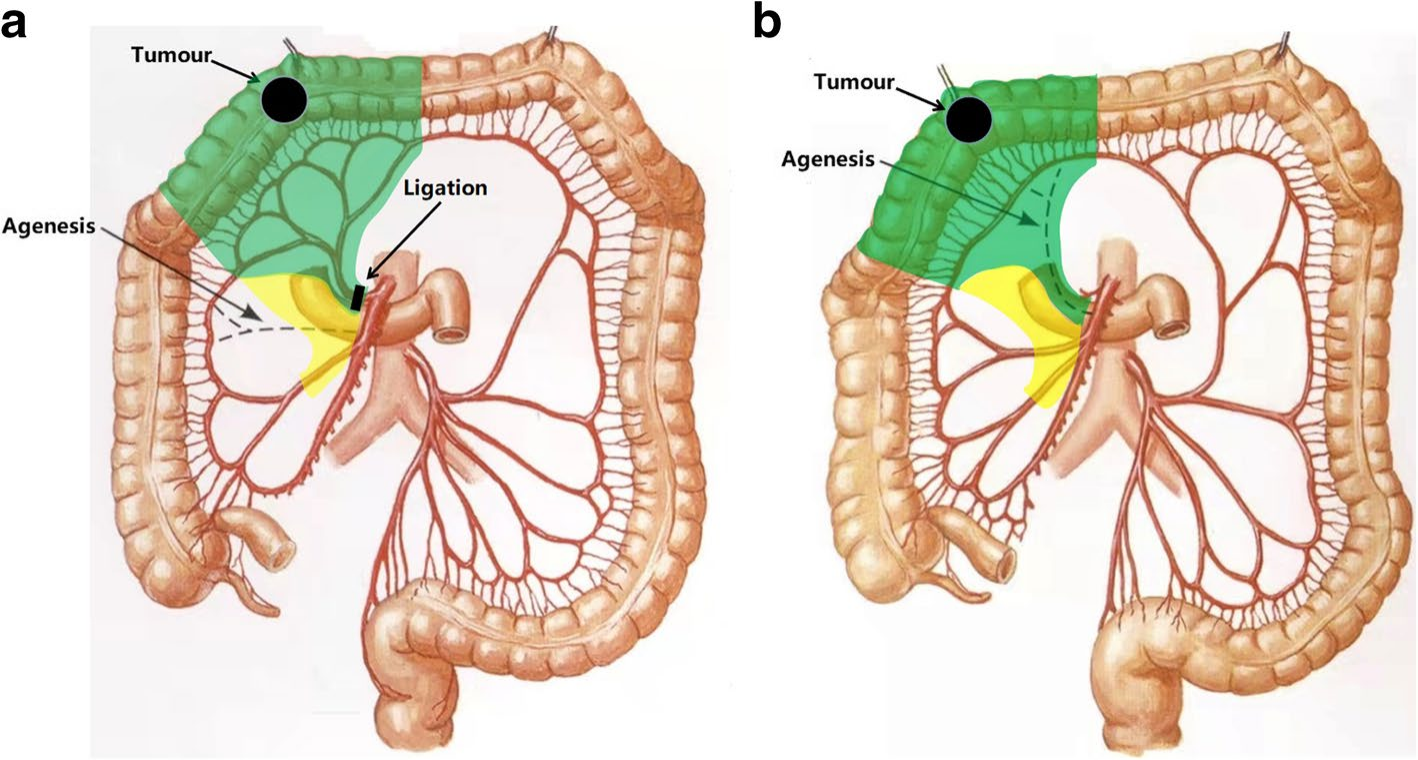
Vascular ligation locations and surgical resection ranges of our procedure for the two common patterns of agenesis. **A** Absence of RCA. **B** Absence of MCA. Anatomical pictures are from the *Atlas of Human Anatomy* [2]

The total operating time was 158 min, and the estimated blood loss was 40 ml. Pathological examination confirmed 2 apical lymph node metastases; among them, one apical lymph node metastasis was in group No.203 (Fig. 5, yellow circle). The pathologic staging was pT3N1M0 (stage IIIB). Our patient recovered uneventfully after surgery and received adjuvant postoperative chemotherapy. Because of the preservation of the ileocecal junction and part of the ascending colon, she kept a stable condition with no long-term diarrhea and water-electrolyte imbalance after the operation. There was also no evidence of tumor recurrence after more than 3 years of follow-up. This operation was also performed in 8 other patients. The median operative time was 160 min (range, 140–185 min), the average number of lymph node retrieval was 30 (range, 25–39), the average number of apical lymph node (No.203, No.213, and No.223) retrieval was 5.9 (range, 0–11), and all patients did not experience long-term diarrhea, water-electrolyte imbalance, and other Clavien–Dindo grade III or greater postoperative complications (Table 1). The right colon and transverse colon had highly variable arterial patterns [2–5], such as the absence of some branches, common trunks, and supernumerary branches. To better perform our procedure, we summarized several vascular ligation locations and surgical resection ranges in several common arterial patterns. The common variations included common trunks of RCA and MCA, common trunks of RCA and IA, and the absence of RCA or MCA. Vascular ligation locations and surgical resection ranges of our procedure for the two patterns of common trunks were shown in Fig. 6. Vascular ligation locations and surgical resection ranges of our procedure for the two patterns of agenesis are shown in Fig. 7.

**Table 1:** Surgical and pathological outcomes of 9 patients

Case	Operative time (min)	LNM/LNR	LNM/LNR (apical)			Complication	Recurrence
			No. 203	No. 213	No. 223		
1	158	3/30	1/3	0/0	1/5	None	None
2	185	6/31	0/2	0/0	1/4	None	None
3	150	0/27	0/0	0/0	0/0	None	None
4	170	7/39	1/3	0/2	1/6	None	None
5	140	0/28	0/0	0/1	0/2	None	None
6	165	1/25	0/1	0/0	0/4	None	None
7	160	3/29	0/2	0/1	0/4	None	None
8	145	5/33	0/2	1/2	1/6	None	None
9	164	0/28	0/0	0/0	0/3	None	None

*LNM* lymph node metastasis, *LNR* lymph node retrieval

## Discussion

In recent years, many studies [6–9] have been focused on the comparison of segmental colectomy and right hemicolectomy. But there is still no consensus on the optimal operation for TCC. Laparoscopic segmental colectomy with extensive D3 lymph node dissection is a less invasive operation on the basis of radical oncological outcomes. It emphasizes an extensive apical lymph node dissection along the superior mesenteric vessels and its main branches but preserves the ileocecal junction and part of the ascending colon (Fig. 1). It combines the advantages of segmental colectomy and right hemicolectomy and gives consideration to oncological and functional outcomes. The 5-year survival rate of TCC patients is 28–50%; it is obviously poorer than that of other colorectal cancer due to more extensive lymph node metastasis [3]. Because of the variation of arterial and venous anatomy, radical operation for TCC is also considered more difficult than other colorectal cancers. One difficulty comes from lymph node dissection around the middle colic vessels; another difficulty comes from the adjacent anatomical structures of the transverse mesocolon such as the duodenum, pancreas, spleen, and the superior mesenteric vessels. At present, the main standardized surgical concepts for TCC include high ligation of the middle colic vessels, D3 lymph node dissection, complete mesocolic excision, segmental colectomy, and extended colectomy (right hemicolectomy). As we know, the number of harvested lymph nodes is considered as a vital symbol of surgical quality and prognosis for colorectal cancer. For better oncological outcomes, extended right hemicolectomy is the preference of many surgeons for a TCC, especially for a right TCC. It includes ligation of the ileocolic, right colic, and middle colic vessels [1, 3]. Compare with extended right hemicolectomy, segmental colectomy only includes ligation of the middle colic vessels. Because of a low number of harvested lymph nodes, segmental colectomy with D3 lymph node dissection is even considered as a less radical surgical procedure by many people. From the perspective of D3 lymph node dissection, inadequate dissection of apical lymph nodes may be the main reason. Apical lymph node metastasis is associated with a poor prognosis [10]. However, segmental colectomy is a less invasive operation from the view of surgical safety; it preserves a longer length of the normal colon and the ileocecal junction. The preservation of the ileocecal junction could reduce the hydro-electrolytic loss and difficulty in adapting to the postoperative diet [11].

## Conclusions

Our procedure combines the advantages of traditional colectomy and right hemicolectomy and gives consideration to oncological and functional outcomes. It is highly plausible from the perspective of surgical oncology. We think it may be an optimal choice for TCC, especially for TCC with a very long transverse colon and preoperative diagnosis of lymph node metastasis.

## Supplementary Information

**Additional file 1.** Supplementary video.

## Data Availability

The datasets used and/or analyzed during the current study are available from the corresponding author on reasonable request.

## Abbreviations

TCC: Transverse colon cancer
IA: Ileocolic artery
IV: Ileocolic vein
ARCA: Accessory right colic artery
RCA: Right colonic artery
RCV: Right colonic vein
GTH: Gastrocolic trunk of Henle
MCA: Middle colic artery
RGA: Right gastroepiploic artery
RGV: Right gastroepiploic vein
AIPDV: Anterior inferior pancreaticoduodenal vein
